# Comparative antibiogram of coagulase-negative *Staphylococci* (CNS) associated with subclinical and clinical mastitis in dairy cows

**DOI:** 10.14202/vetworld.2015.421-426

**Published:** 2015-03-28

**Authors:** B. K. Bansal, D. K. Gupta, T. A. Shafi, S. Sharma

**Affiliations:** Department of Veterinary Medicine, Guru Angad Dev Veterinary and Animal Sciences University, Ludhiana - 1410 04, Punjab, India

**Keywords:** antibiotic, coagulase-negative staphylococci, cows, culture and mastitis

## Abstract

**Aim:**

The present study was planned to determine the *in vitro* antibiotic susceptibility of coagulase-negative *Staphylococci* (CNS) strains isolated from clinical and subclinical cases of mastitis in dairy cows. Antibiotic sensitivity profile will be helpful to recommend early therapy at the field level prior to availability of CST results.

**Materials and Methods:**

The milk samples from cases of clinical mastitis received in Mastitis Laboratory, Guru Angad Dev Veterinary and Animal Sciences University, Ludhiana and those of subclinical mastitis collected during routine screening of state dairy farms, were subjected to microbial culture. Identification of CNS organisms was done by standard biochemical tests. Antibiotic sensitivity testing, based on 30 antibiotics belonging to 12 groups, was done on 58 randomly selected CNS isolates (clinical isolates: 41, subclinical isolates: 17).

**Results:**

Isolates were highly susceptible to chloramphenicol (98.3%), gentamicin (93.1%), streptomycin (91.4%), linezolid (91.4%), ceftixozime (87.9%), cloxacillin (86.2%), clotrimazole (86.2%), bacitracin (86.2%), enrofloxacin (84.5%) and ceftrioxone + tazobactum (70.7%), while resistance was observed against amoxicillin (77.6%), penicillin (75.9%), ampicillin (74.1%) and cefoperazone (51.7%). Overall, isolates from clinical cases of mastitis had a higher resistance than subclinical isolates.

**Conclusion:**

CNS isolates were susceptible to chloramphenicol, gentamicin and streptomycin, while higher resistance was recorded against routinely used penicillin group.

## Introduction

Mastitis, the inflammation of mammary glands, is a multi-factorial disease that imposes serious economic losses to the farmers and the dairy industry [1]. Mastitis is one of the most costly diseases of dairy cattle resulting in the reduction of milk yield and quality [2]. Annual economic losses due to subclinical mastitis and clinical mastitis in India have been estimated to be Rs. 4151.1 and Rs. 3014.4 crores, respectively with a total of Rs. 7165.5 crores [3]. Mastitis can occur either in subclinical or clinical form and etiology has been classified as contagious and environmental pathogens.

Mastitis caused by coagulase-negative staphylococci (CNS) has been primarily considered subclinical, with only a slight increase in milk somatic cell count (SCC) [4]. In 1985, only 15 CNS species were recognized and since then, many new species of *Staphylococcus* have been discovered [5]. Some important CNS species are *Staphylococcus chromogenes*, *Staphylococcus* *sciuri, Staphylococcus* *capitis*, *Staphylococcus* *hominis, Staphylococcus* *epidermidis, Staphylococcus*, *simulans, Staphylococcus* *haemolyticus* and *Staphylococcus Xylosus*. However, in the recent studies, CNS species have been recovered from cases of subclinical as well as clinical mastitis [6]. Though CNS species are less virulent than *Staphylococcus aureus*, these generally exhibit higher antimicrobial resistance, and more often show multi-antimicrobial resistance [7]. The importance of CNS as the predominant pathogens of mastitis has been recognized in the recent years [8,9]. The understanding on Staphylococcal mastitis is fairly complicated by the large number of species and the heterogeneity within the group.

In India, there have been only limited studies of drug resistance patterns of CNS. Therefore, the present study was planned to determine the *in vitro* antibiotic susceptibility of CNS strains isolated from clinical and subclinical cases of mastitis in dairy cows. Antibiotic susceptibility profile of CNS isolates, reported in this study, will be helpful to recommend early therapy at the field level prior to availability of CST results.

## Materials and Methods

### Ethical approval

Not necessary.

### Collection of milk samples

The milk samples from cases of mastitis, submitted in Mastitis Laboratory of Guru Angad Dev Veterinary and Animal Sciences University, Ludhiana, were subjected to bacteriological culture. Besides, milk samples collected from apparently healthy cows during routine screening of state dairy farms for subclinical mastitis, were subjected to microbial culture. Some of the composite milk samples submitted as a measure of herd screening were also included in the study. A total of 859 milk samples from 270 cows comprising of 157 mastitic cows and 113 apparently healthy cows were used for bacteriological culture.

### Isolation and biochemical characterization of CNS

Procedures described by National Mastitis Council USA [10] were followed for culture of milk samples and identification of mastitis pathogens. The milk samples brought to the laboratory were mixed thoroughly and streaked on Blood Agar plates. Each plate was divided into four equal parts marked for individual quarters, and 0.05 ml of quarter milk sample was streaked with sterilized Platinum loop. The plates were incubated aerobically at 37°C and examined after 18-24 h for the presence of any bacterial growth. The representative colonies of the microorganisms were isolated and maintained by streaking onto fresh brain heart infusion agar plates. The organisms were identified on the basis of colony characteristics on blood agar, Gram staining, clumping factor, growth characteristics on mannitol salt agar, DNase agar and Baird parker agar and tube coagulase test.

### Culture sensitivity testing

A total of 30 antibiotics belonging to 12 groups were used to study antibiotic sensitivity profile of CNS isolates. Susceptibility of bacterial isolates from mastitic milk to anti-microbials was subjected using the disc diffusion susceptibility test (Kirby Bauer method) [11]. Briefly, a fresh colony of the isolates was transferred to a tube containing 5 ml nutrient broth. The mixture was incubated at 37°C until light visible turbidity appeared; this was compared with the McFarland 0.5 turbidity standard. The suspension of test organism was streaked over the surface of Muller Hinton agar plates using a sterile disposable cotton swab. Commercially available antibiotics discs (Hi-Media) were firmly placed on plates by means of sterile forceps and plates were incubated for 24 h at 37°C. The diameters of growth-inhibition were measured in mm and reported as, susceptible, intermediate, and resistant, as per CSLI guidelines.

## Results and Discussion

The present study was envisaged to identify the CNS in the mastitic milk samples, to reveal their antibiotic sensitivity pattern and to carry out rationale treatment. Of the 346 milk samples from 113 apparently healthy cows, 129 bacterial isolates of different microorganisms were recovered, and CNS were the most frequently recovered bacterial species accounting for (29.5%) of all isolates. In the case of clinically mastitis cows (n=157), of the 513 milk samples, 180 bacterial isolates of different microorganisms were recovered and CNS were the most frequently recovered bacterial species accounting for (34.4%) of all isolates. In the present study, the prevalence of CNS in clinical (34.44%) mastitis was higher than subclinical (29.69%) mastitis which is contrary to the findings of Persson Waller *et al*. (9) and Pyorala and Taponen [7], while our findings are in agreement with studies conducted in Finland [12], Germany [13], Italy [14] and Korea [15].

In many modern dairy herds, opportunistic bacteria such as CNS are frequently associated with bovine mastitis, and hence could be described as emerging pathogens [7]. CNS possesses traits of both environmental and contagious pathogens. Although they are not normal inhabitants of the mammary gland, they commonly colonize the skin of the teat, the teat end, teat injuries, and the hands of milkers. Therefore, they can spread mechanically and present major risks for cows with teat-end injuries [16]. CNS has also been the most common finding [17,18] but *S. aureus* has been the most commonly encountered pathogen in a number of studies from other countries [9,19-21].

The increasing role of CNS as a cause of subclinical and clinical mastitis and emergence of antimicrobial resistance in them is becoming a matter of concern for the dairy industry. In the present study, 58 CNS isolates selected randomly from cases of clinical (n=41) and subclinical (n=17) were subjected to culture sensitivity testing. A total of 30 antibiotics belonging to 12 groups were used to study the drug resistance pattern. Sensitivity of the CNS isolates to antibiotic agents (along with their concentration) is presented in Table-1. Since the CSLI guidelines do not provide interpretive criteria for some of the mastitis pathogens/antimicrobial combinations, whenever guidelines for testing and breakpoints were not available for a specific pathogen, guidelines for other pathogens/antimicrobial combinations of the same group were used. Such a method is commonly used by commercial laboratories and has been reported in literature [12,22]. Thus, the breakpoints for ampicillin were used for amoxicillin and those for oxacillin were used for cloxacillin.

**Table-1:** Antibiotic sensitivity test of CNS against different antibiotics.

Group	Antibiotic	Breakpoint (mm)		*n* (%)		

				CM	SCM	Total
β-Lactams	Penicillin-G	S	≥29	6 (14.63)	8 (47.1)	14 (24.14)
		I	NA	NA	NA	NA
		R	≤28	35 (85.37)	9 (52.9)	44 (75.86)
	Amoxycillin+Clavulanate	S	≥20	23 (56.1)	10 (58.8)	33 (56.90)
		I	NA	NA	NA	NA
		R	≤19	18 (43.9)	7 (41.2)	25 (43.10)
	Ampicillin	S	≥29	7 (17.1)	8 (47.1)	15 (25.86)
		I	NA	NA	NA	NA
		R	≤28	34 (82.9)	9 (52.9)	43 (74.14)
	Amoxycillin+Sulbactam	S	≥15	23 (56.1)	12 (70.6)	35 (60.34)
		I	12-14	3 (7.3)	2 (11.8)	5 (8.62)
		R	≤11	15 (36.6)	3 (17.6)	18 (31.03)
	Amoxycillin	S	≥29	5 (12.2)	8 (47.1)	13 (22.41)
		I	NA	NA	NA	NA
		R	≤28	36 (87.8)	9 (52.9)	45 (77.59)
	Cloxacillin	S	≥13	33 (80.5)	17 (100.0)	50 (86.21)
		I	11-12	2 (4.9)	0 (0.0)	2 (3.45)
		R	≤10	6 (14.6)	0 (0.0)	6 (10.34)
	Oxacillin	S	≥13	37 (90.2)	15 (88.2)	52 (89.66)
		I	11-12	0 (0.0)	0 (0.0)	0 (0.00)
		R	≤10	4 (9.8)	2 (11.8)	6 (10.34)
	Ceftrioxone+Sulbactam	S	≥21	22 (53.7)	8 (47.06)	30 (51.72)
		I	14-20	18 (43.9)	9 (52.94)	27 (46.55)
		R	≤13	1 (2.4)	0 (0.00)	1 (1.72)
	Ceftrioxone+Tazobactum	S	≥21	29 (70.7)	12 (70.6)	41 (70.69)
		I	14-20	12 (29.3)	5 (29.4)	17 (29.31)
		R	≤13	0 (0.0)	0 (0.00)	0 (0.00)
	Ceftrioxone	S	≥21	19 (46.3)	8 (47.1)	27 (46.55)
		I	14-20	21 (51.2)	9 (52.9)	30 (51.72)
		R	≤13	1 (2.4)	0 (0.00)	1 (1.72)
	Ceftazidine	S	≥18	27 (65.9)	11 (64.7)	38 (65.52)
		I	15-17	5 (12.2)	4 (23.5)	9 (15.52)
		R	≤14	9 (21.9)	2 (11.8)	11 (18.97)
	Cefoperazone	S	≥24	19 (46.3)	9 (52.9)	28 (48.28)
		I	NA	NA	NA	NA
		R	≤23	22 (53.7)	8 (47.1)	30 (51.72)
	Ceftixozime	S	≥20	35 (85.4)	16 (94.1)	51 (87.93)
		I	15-19	4 (9.8)	0 (0.0)	4 (6.90)
		R	≤14	2 (4.9)	1 (5.9)	3 (5.17)
	Ceforoxime	S	≥18	36 (87.8)	16 (94.1)	52 (89.66)
		I	15-17	2 (4.9)	1 (5.9)	3 (5.17)
		R	≤14	3 (7.3)	0 (0.0)	3 (5.17)
Aminogylcosides	Gentamicin	S	≥15	37 (90.2)	17 (100.0)	54 (93.10)
		I	12-14	4 (9.8)	0 (0.0)	4 (6.90)
		R	≤11	0 (0.0)	0 (0.0)	0 (0.00)
	Neomycin	S	≥15	31 (75.6)	15 (88.2)	46 (79.31)
		I	13-14	3 (7.3)	1 (5.9)	4 (6.90)
		R	≤12	7 (17.1)	1 (5.9)	8 (13.79)
	Streptomycin	S	≥14	38 (92.7)	15 (88.2)	53 (91.38)
		I	NA	NA	NA	NA
		R	≤13	3 (7.3)	2 (11.8)	5 (8.62)
	Amikacin	S	≥17	39 (95.1)	15 (88.2)	54 (93.10)
		I	15-16	1 (2.4)	2 (11.8)	3 (5.17)
		R	≤14	1 (2.4)	0 (0.0)	1 (1.72)
Floroquinolones	Enrofloxacin	S	≥22	33 (80.5)	16 (94.1)	49 (84.48)
		I	18-21	5 (12.2)	0 (0.0)	5 (8.62)
		R	≤17	3 (7.3)	1 (5.9)	4 (6.90)
	Ciprofloxacin	S	≥21	36 (87.8)	17 (100.0)	53 (91.38)
		I	16-20	3 (7.3)	0 (0.0)	3 (5.17)
		R	≤15	2 (4.9)	0 (0.0)	2 (3.45)
	Moxifloxacin	S	≥24	32 (78.1)	15 (88.2)	47 (81.03)
		I	21-23	3 (7.3)	2 (11.8)	5 (8.62)
		R	≤20	6 (14.6)	0 (0.0)	6 (10.34)
Sulphonamide	Clotrimazole	S	≥16	33 (80.5)	17 (100.0)	50 (86.21)
		I	11-15	2 (4.9)	0 (0.0)	2 (3.45)
		R	≤10	6 (14.6)	0 (0.0)	6 (10.34)
Macrolide	Erythromycin	S	≥23	28 (68.3)	11 (64.7)	39 (67.24)
		I	14-22	11 (26.8)	6 (35.3)	17 (29.31)
		R	≤13	2 (4.9)	0 (0.0)	2 (3.45)
Tetracycline	Tetracycline	S	≥19	29 (70.7)	10 (58.8)	39 (67.24)
		I	15-18	6 (14.6)	2 (11.8)	8 (13.79)
		R	≤14	6 (14.6)	5 (29.4)	11 (18.97)
Lincosamide	Clindamycin	S	≥21	31 (75.6)	12 (70.6)	43 (74.14)
		I	15-20	7 (17.1)	3 (17.6)	10 (17.24)
		R	≤14	3 (7.3)	2 (11.8)	5 (8.62)
Oxazolidinone	Linezolid	S	≥21	37 (90.2)	16 (94.1)	53 (91.38)
		I	NA	NA	NA	NA
		R	≤20	4 (9.8)	1 (5.9)	5 (8.62)
Phenicols	Chloramphenicol	S	≥18	40 (97.6)	17 (100.0)	57 (98.28)
		I	13-17	1 (2.4)	0 (0.0)	1 (1.72)
		R	≤12	0 (0.0)	0 (0.0)	0 (0.00)
Aminocoumarine	Novobiocin	S	≥22	32 (78.1)	15 (88.2)	47 (81.03)
		I	18-21	NA	NA	NA
		R	≤17	9 (22.0)	2 (11.8)	11 (18.97)
Glycopeptide	Teicoplanin	S	≥14	31 (75.6)	15 (88.2)	46 (79.31)
		I	11-13	8 (19.5)	2 (11.8)	10 (17.24)
		R	≤10	2 (4.9)	0 (0.0)	2 (3.45)
Miscellaneous	Bacitracin	S	≥13	33 (80.5)	17 (100.0)	50 (86.21)
		I	9-12	2 (4.9)	0 (0.0)	2 (3.45)
		R	≤8	6 (14.6)	0 (0.0)	6 (10.34)

Breakpoint depicts Zone of inhibition of antibiotics in mm. S=Sensitive, I=Intermediate, R=Resistant, NA=Not Aplicable, A=Ampicillin (10 mcg), AM=Amoxycillin (10 mcg), AS=Amoxycillin+sulbactam (30/15 mcg), AC=Amoycillin+clavulanate (30 mcg), P=Penicillin (2 units), OX=Oxacillin (1 mcg) COX=Cloxacillin (10 mcg), CI=Ceftrioxone (10 mcg), CIS=Ceftrioxone+sulbactam (30/15 mcg), CIT=Ceftrioxone+tazobactum (30/10 mcg), CAZ=Ceftazidine (30 mcg), CPZ=Cefoperazone (75 mcg), CXM=Ceforoxime (30 mcg), CZX=Ceftixozime (30 mcg), EX=Enrofloxacin (10 mcg), CIP=Ciproflaxacin (5 mcg), MO=Moxifloxacin (5 mcg), G=Gentamicin (10 mcg), AK=Amikacin (30 mcg), S=Streptomycin (25 mcg), N=Neomycin (30 mcg), COT=Clotrimazole (25 mcg), E=Erythromycin (10 mcg), T=Tetracycline (10 mcg), CD=Clindamycin (2 mcg), Lz=Linezolid (30 mcg), C=Chloramphenicol (30 mcg), Tei=Teicoplanin (30 mcg), B=Bacitracin (10 mcg), NV=Novobiocin (30 mcg)

It was noted that most of the isolates were highly susceptible to chloramphenicol (98.3%), gentamicin (93.1%), streptomycin (91.4%), linezolid (91.4%), ceftixozime (87.9%), cloxacillin (86.2%), clotrimazole (86.2%), bacitracin (86.2%), enrofloxacin (84.5%) and ceftrioxone + tazobactum (70.7%) (Table-1). CNS species were highly sensitive to all the cephalosporins except cefoperazone (48.3%) which was found to be moderately efficacious.

Among β-Lactams, cloxacillin and potentiated penicillins were highly sensitive while resistance was shown against penicillin, ampicillin, and amoxicillin. The rate of penicillin resistance (75.9%) observed in this study is much higher than those reported in other countries such as Korea (52.9%), Switzerland (31%), Finland (32%), the USA (22.1%) and Turkey (58.3%) [12,23,24]. Resistance in staphylococci has been associated with the production of β-lactamases and low-affinity penicillin-binding protein, PBP2a [25]. The presence of β-lactamases in CNS has been observed both in human and veterinary isolates. Archer and Scott [26] reported that 89% of CNS isolated from humans harbored inducible β-lactamase. β-Lactamase production has been reported in 21-84% of CNS isolated from dairy cows with mastitis [22,27]. More recently, Taponen *et al*. [5] reported presence of β lactamases in 19% of CNS that caused mastitis in lactating dairy cows.

Among aminogylcosides, gentamicin (93%) was the most sensitive drug compared to other group members. Among floroquinolones, enrofloxacin (84.5%) and ciproflaxacin (91.4%) were highly sensitive. High sensitivity of CNS to enrofloxacin and gentamicin has also been reported in previous studies [28-31].

Macrolides and lincosamides were found to be highly sensitive in the present study, and earlier studies have demonstrated resistance of CNS to macrolides and lincosamides to be 6-7% in Germany [32], and 92% in northern Jordan [31]. Moderate resistance shown by tetracyclines (19%) in the present study, is comparable with that reported in Finland (9%) [12], while in contrast Alekish *et al*. [31] and Mahami *et al*. [33] reported 100% resistance to tetracycline.

Resistance to antimicrobial agents in bacteria may occur due to a spontaneous mutation or acquired via plasmids, transposons and integrons from resistant bacteria. Overall, antibiotic resistance in this study was considerably lower in isolates from subclinical mastitis in comparison to isolates from clinical mastitis (Table-1). Higher resistance among isolates from clinical mastitis cases could be attributed to indiscriminate use of antibiotics in clinical cases without following proper dosage regimen [34]. Besides, recurrent nature of the clinical mastitis promotes repetition of the antibiotics for which the pathogen has acquired the resistance at first instance. Thus caution must be practiced while treating clinical mastitis case, as there is narrow choice of antibiotics for treatment before CST results are available.

## Conclusion

CNS isolates are susceptible to chloramphenicol, gentamicin, and streptomycin while higher resistance was recorded against routinely used penicillin group. Overall, isolates from clinical cases of mastitis had a higher resistance than subclinical isolates.

## Authors’ Contribution

BKB supervised the research. TAS and DKG were involved with collection and processing of samples in the laboratory. TAS and SS participated in draft and revision of the manuscript. All authors read and approved the final manuscript.
